# Cationic/Anionic Poly(p-Phenylene Oxide) Membranes: Preparation and Electrodialysis Performance for Nickel Recovery from Industrial Effluents

**DOI:** 10.3390/membranes14120268

**Published:** 2024-12-11

**Authors:** Fabrício Wilbert, Joana Farias Corte, Felipe Tiago do Nascimento, Vanusca Dalosto Jahno, Marco Antônio Siqueira Rodrigues, Fabrício Celso, Salatiel W. da Silva, Andrea Moura Bernardes

**Affiliations:** 1 Laboratório Aquário, Novo Hamburgo, Feevale University, Novo Hamburgo 93525-075, Brazil; fabricio.wilbert@gmail.com (F.W.); joanacortef@gmail.com (J.F.C.); eng.felipetn@gmail.com (F.T.d.N.); vanusca@feevale.br (V.D.J.); marcor@feevale.br (M.A.S.R.); 2 Development Technology Center, Federal University of Pelotas, Pelotas 96160-000, Brazil; fcelso1976@gmail.com; 3 Instituto de Pesquisas Hidráulicas, Programa de Pós-Graduação em Engenharia de Recursos Hídricos e Saneamento Ambiental—Federal University of Rio Grande do Sul, Porto Alegre 91501-970, Brazil; salatiel.silva@ufrgs.br; 4 Programa de Pós-Graduação em Engenharia de Minas, Metalúrgica e de Materiais—Federal University of Rio Grande do Sul, Porto Alegre 91501-970, Brazil

**Keywords:** electrodialysis, ion-selective membranes, PPO membranes, nickel recovery

## Abstract

Electrodialysis (ED) has already been applied to recover nickel in galvanizing processes, allowing nickel recovery and the production of a treated effluent with demineralized water quality. However, the growth in ED use is still limited by the production and commercialization of ion-selective membranes, currently limited to a few large companies. Therefore, this paper presents the development of homogeneous cationic and anionic membranes made from poly(2,6-dimethyl-1,4-phenylene oxide) (PPO) for ED use. Cationic membranes were prepared by the sulfonation reaction of PPO under different experimental conditions (PPO:H_2_SO_4_ molar ratio and reaction time). Anionic membranes were prepared by the bromination reaction of PPO, followed by the amination reaction. The membranes were characterized for their chemical and electrochemical properties, including ion exchange capacity, conductivity, thermal stability, and surface morphology. The optimal conditions for cationic membrane sulfonation were achieved with a 1:4.4 PPO:H_2_SO_4_ molar ratio, and a reaction time of 0.5 h. For anionic membranes, the best results were obtained with bromination, with a PPO:NBS (N-Bromosuccinimide) molar ratio of 1:0.5, followed by 14 days of amination. Overall, 91.8% chloride, 90.9% sulfate, and 85.5% nickel ion extraction was achieved, highlighting PPO as a promising polymer for the development of anionic and cationic ion-selective membranes for ED.

## 1. Introduction

Despite its abundant water resources, Brazil faces severe pollution issues in several hydrographic basins, especially in urban areas. This pollution compromises drinking water sources, affecting their availability for human consumption. Conventional water and effluent treatment methods are often inefficient and generate hazardous wastes. Therefore, there is a growing need for research focused on reducing water contamination and improving the reuse of industrial effluents.

Electroplating is a process that has a wide and diverse range of applications in various industries, including automobiles, household appliances, civil construction, electronics, petrochemicals, and furniture, among others. In this process, a surface is covered with metal to protect it from corrosion or to give an esthetic finish. However, the electroplating process generates effluents containing metal ions, which could be recovered and reused in the production process. The most commonly used treatment for these effluents is a physical–chemical treatment. This treatment generates sludge, which is classified as hazardous, and causes serious environmental damage if not disposed of in accordance with environmental standards [1]. From both an environmental and an economical point of view, it is important to employ a treatment process for these effluents that allows the recovery of metal ions. Given this scenario, electrodialysis (ED) can be used in the treatment of electroplating effluents with two objectives: (i) to promote the reuse of the treated effluent as process rinsing water, and (ii) to allow the reuse of the concentrated effluent as a replenisher for the electroplating bath. Thus, it provides the application of two important sustainability concepts: zero effluent disposal and circular economy in the industry [2,3].

Considering that the use of ED and its related processes is a way to reduce waste and to recover nickel from plating effluents, several studies have been developed applying an electro-electrodialysis system [2], electrodeposition–electrodialysis [4], and electrodialysis [5,6,7]. These studies were carried out with a DuPont Nafion^®^ cationic membrane and an IONSEP^®^ anionic membrane. The results demonstrated the economical and operational viability of ED in this application. However, the scaling-up of this technology to industrial wastewater treatment plants is still very incipient. To improve the use of ED processes to wastewater treatment, it is important to develop a low-cost technology to produce ion-exchange membranes [8].

Regarding the industrial production of membranes, the use of a single polymer as a precursor to obtain both anionic and cationic membranes greatly simplifies the entire production logistics. Poly (2,6-dimethyl-1,4-phenylene oxide) (PPO) has excellent physicochemical properties and a versatile chemical structure, allowing it to be modified to produce a cationic and an anionic membrane [9,10,11]. The synthesis of PPO was reported in 1951 [12], and since then, PPO has proven to be a valuable polymer for various applications, due to its high glass transition temperature and excellent mechanical resistance and dimensional stability, among other engineering properties. One notable application of PPO is in the separation of gas mixtures, where it has achieved gas permeability and selectivity results similar to or even superior to those of the Nafion membrane in some cases [13].

In the case of cationic membranes, PPO sulfonation has been investigated by several authors [14,15,16], as sulfonated PPO (SPPO) can be used to obtain cation-exchange membranes, opening several applications for this polymer, such as in energy production systems (fuel cells), battery separators, semiconductors, and the deionization of brackish water [17]. In the past, different chemicals have been used as PPO sulfonation agents. They include concentrated sulfuric acid, fuming sulfuric acid, and chlorosulfonic acid (CSA) [14]. Concentrated sulfuric acid was considered difficult to use, as polymer stability was compromised depending on reaction conditions, even causing polymer degradation [11,18,19]. Given these results, chlorosulfonic acid was recognized as an adequate sulfonating agent, and the vast majority of publications since then have used chlorosulfonic acid as a sulfonating agent of PPO [16,19,20]. However, CSA is an expensive reagent and difficult to acquire in some countries. Therefore, one contribution of this work was to investigate the parameters to use concentrated sulfuric acid as a sulfonating agent of PPO, with the aim of preparing cationic membranes for application in electrodialysis processes.

Regarding anionic membrane production, PPO membranes were also investigated for fuel cells, but the literature [21] points out structural problems and membrane fragility. In this work, the PPO bromination process was carried out with N-Bromosuccinimide (NBS) and subsequent amination with trimethylamine (TMA), investigating the influence of the NBS:PPO molar fraction on the bromination of PPO to form brominated PPO (BPPO). This work contribution was the evaluation of using a support fabric in the manufacture of anionic membranes, to provide the necessary structural resistance to the membranes that will be applied in ED systems.

Thus, the objective of this research was to produce cationic and anionic membranes from the same polymer (PPO) with enough stability to be applied in electrodialysis. The produced membranes were then evaluated for nickel recovery from an electroplating industrial effluent.

## 2. Materials and Methods

### 2.1. Materials

The poly(p-phenylene oxide) used in this work was Sabic Innovative Plastics Noryl PPO Powder 646 thermoplastic polymer. Chloroform, sulfuric acid, sodium hydroxide, hydrochloric acid n-methyl-2-pyrrolidone (NMP), chlorobenzene, n-bromosuccinimide (NBS), methanol, benzoyl peroxide, trimethylamine, and sodium sulfate were of analytical grade and used as received. Polyester or polyamide mesh was used as reinforcement for the anionic membranes to ensure good structural properties.

For the comparison of the results, commercial ion-selective membranes were also evaluated. The membranes have a Chinese origin (IONSEP-HC-C and IONSEP-HC-A, Hangzhou Iontech Environmental Technology Co., Hangzhou, China) and are sold in Brazil by the company Hidrodex Engenharia Ltd. (Rua Dona Maria de Barros, Brazil) under the trade names HDX 100 (cationic) and HDX 200 (anionic). Both membranes are heterogeneous, contain ion-exchange resin and a reinforcement woven [22], were used as an industrial reference, since they are employed in industrial applications by the supplier, and have been widely studied by our research group [22,23,24,25]. The characteristics of the commercial cation- and the anion-exchange membranes that were used in this work are given in Table 1 [22].

The nickel electroplating industrial effluent was obtained from a company located in the State of Rio Grande do Sul, Brazil. The effluent was collected from the electroplating production line, specifically from the first rinsing water after the nickel electroplating bath. The rinsing water depletes salts dragged out from one bath to another, which negatively affect the coating obtained by electrodeposition. The characteristics of this effluent are presented in Table 2. It was fed into the central compartment of the ED cell for treatment. Nickel concentrations were determined by Flame Atomic Absorption Spectrophotometry (using an analytical Varian FS-240 Spectrometer). Chloride and sulfate ion analyses were performed by Ion Chromatography (DIONEX ICS-3000). According to the literature [26] for the NiSO_4_ solution, at the initial pH around 4.4, SO_4_^2−^ is the predominant anionic species. In addition to this species, the HSO_4_^−^ ion will also be present and, in a lower concentration, a nickel–sulfate complex [Ni(SO_4_)_2_]^2−^ may also be present.

In all experiments, a conductometer (8361 AZ Instrument) was used to measure the electrical conductivity of the samples. A pH meter (pH-TEC PHS-3B) was used to measure pH levels.

### 2.2. Membrane Preparation

#### 2.2.1. Cationic Membranes

The PPO sulfonation reaction began with the dissolution of PPO powder in chloroform at 55 °C, followed by the dropwise addition of sulfuric acid under a nitrogen atmosphere. Different PPO:H_2_SO_4_ molar ratios and reaction times were evaluated. The resulting sulfonated PPO (SPPO) was precipitated in cold deionized water (10 °C) under mechanical stirring. Once obtained, the SPPO flakes were washed in deionized water at room temperature to remove residual sulfuric acid, followed by ion exchange in a 0.5 M sodium hydroxide solution to prevent SPPO degradation during subsequent drying at 60 °C for 96 h [27,28]. To obtain cationic membranes, the dried SPPO flakes were dissolved in NMP at 80 °C under mechanical stirring. The resulting solutions were cast over a glass plate and placed in an oven for 24 h at 60 °C [29].

#### 2.2.2. Anionic Membranes

The PPO bromination reaction began with the dissolution of PPO powder in chlorobenzene at 132 °C, followed by the addition of benzoyl peroxide as an initiator, and NBS as the bromination agent under a nitrogen atmosphere. After 2 h, the reaction was terminated by precipitation in methanol under mechanical stirring. The resulting brominated PPO (BPPO) was then dried in an oven at 60 °C for 48 h [30,31]. To obtain anionic membranes, the dried BPPO granules were dissolved in chloroform at room temperature for 30 min under mechanical stirring. The resulting solutions were cast over a reinforcing woven (polyester or polyamide mesh) placed on a glass plate and left to dry for 48 h at room temperature (25 °C). The amination of BPPO membranes was carried out by immersing the membranes in trimethylamine for 14 days [32].

### 2.3. Membrane Characterization

The produced membranes were characterized by evaluating different properties such as ion-exchange capacity, conductivity, thermal stability, and surface morphology.

Ion-exchange capacity (IEC): Ion-exchange capacity (IEC) was determined using an acid–base method and is expressed as milliequivalent per gram of the dry membrane (meq g^−1^ dry membrane) [31,33]. Cationic membrane samples of 1 cm^2^ were weighted and immersed in 1 M HCl solution for 72 h, followed by washing in deionized water and immersion in 1 M NaCl for 24 h. Finally, titration with 0.005 M NaOH was performed in triplicate [30,32]. Anionic membrane samples of 4 cm^2^ were weighted and immersed in 1 M KOH solution for 72 h, followed by washing in deionized water and immersion in 0.02 M HCl for 48 h. Finally, titration with 0.005 M NaOH was performed in triplicate [33]. IEC was then calculated by Equation (1):

(1) $$\mathrm{IEC}\;\left(\mathrm{meq}.{\;g}^{-1}\right)=\frac{V_{\mathrm{sol}}\times {\;M}_{\mathrm{sol}}}{M_{\mathrm{memb}}}$$

where V_sol_ and M_sol_ are, respectively, the volume (mL) and concentration (M) of the solution used for titration, and M_memb_ (g) is the weighted membrane mass.

Sulfonation degree (SD): The sulfonation degree (SD) of the cationic membranes was calculated from the IEC results. The relationship between the degree of sulfonation and the IEC was calculated according to the literature [18,34,35] by Equation (2):

(2) $$\mathrm{SD}\;\left(\%\right)=\frac{{\mathrm{MW}}_{\mathrm{PPO}}\times \;\mathrm{IEC}}{1000-{\mathrm{MW}}_{{\mathrm{SO}}_{3}H}\times \;\mathrm{IEC}}$$

where the value of molar weight (MW) of the monomer unit (PPO) is 120, and the molar weight (MW) of the sulfonic group (SO_3_H) is 81.

Conductivity: Membrane samples of 2 cm in length by 0.5 cm in width were used to measure sample resistivity in the longitudinal direction, in an electrochemical cell connected to a Solartron SI 1260 Impedance/Gain Phase Analyzer with Zplot^®^ software (Version 3.5f). This was operated in frequence sweep mode, in the range from 1 Hz to 1.10^7^ Hz, at 20 °C and 100% relative humidity. The membrane samples were hydrated by soaking in distilled water overnight and placed wet in the measurement cell [36,37,38].

Thermal stability: Thermogravimetric and thermogravimetric derivative analysis (TGA and DTG, respectively) were carried out in a Perkin-Elmer STA-600 (PerkinElmer Company, Waltham, MA, USA) simultaneous thermal analyzer under a nitrogen atmosphere (20 mL/min) to determine the thermal stability of the membranes. The heating rate was 10 °C/min, in the range of 20 °C to 900 °C.

Surface Morphology: Scanning electron microscopy (SEM) images of cationic and anionic membrane surfaces were obtained using a JSM-6510LV JEOL (JEOL Company, Tokio, Japan) scanning electron microscope at 10 kV. All samples were dried and gold-sputtered before analysis. Images are shown with 5000× of magnification.

### 2.4. Electrodialysis Experiments

**Figure 1:**
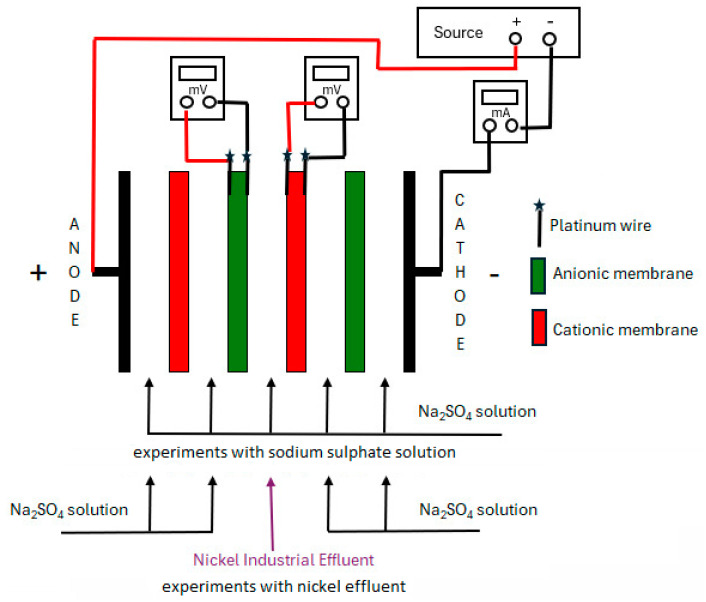
Electrodialysis bench system used for nickel wastewater treatment.

The ED experiments were carried out in a five-compartment bench cell (Figure 1), with four membranes, each with 16 cm^2^ of membrane area. All experiments were conducted using 600 mL of Na_2_SO_4_ solution (0.0123 mol.L^−1^; pH 6.3; 2.95 mS.cm^−1^) in four cell compartments. Nickel industrial effluent (600 mL) was placed in the central compartment of this bench cell. The cell was fed by centrifugal pumps, with a flow rate of 85 L h^−1^ in a batch mode, also ensuring the circulation of the solutions in the reservoirs.

Ti/0.7TiO_2_/0.3RuO_2_ electrodes, in plate format and with an area of 16 cm^2^, were used as cathode and anode. Direct electric current was applied using a Hikari source HF-303S.

ED experiments were carried out with the HDX commercial membranes, as well as with the membranes produced in this work. Before each ED experiment, the membrane samples were immersed in the solutions of the bench cell for 24 h.

The experiments to evaluate the ionic transport through the membranes were carried out with an applied electric current density of 6.25 mA/cm^2^ for 2 h. The transport of ions from the central to the other cell compartments was evaluated by calculating the percent extraction of ions according to Equation (3):

(3) $$\mathrm{PE}\;\left(\%\right)=\frac{C_{i}-C_{f}}{C_{i}}\times 100$$

where C_i_ is the concentration of the considered ion in the central compartment at time zero and C_f_ is the concentration of the considered ion in the central compartment at the final time.

To determine the electrical limiting current density of the membranes, current–voltage curve (CVC) experiments were carried out in the same bench cell, hydrodynamic conditions, and solution concentrations. The current–voltage curves were obtained by gradually increasing the current and measuring the potential drop across the membrane after a stabilization period (2 min) with a voltmeter and by means of two platinum electrodes, each one placed on either side of the membrane, close to the surface. Once the potential drop had been measured, no current was applied for 3 min. The current source was then switched on again, with an increase in current relative to the previously applied current, and the new potential drop across the membrane was again measured after 2 min. The assembly diagram of the system is shown in Figure 1. In some CVC experiments, 600 mL of Na_2_SO_4_ solution (1.75 g.L^−1^) was also used in the central compartment, for better comprehension of the system.

## 3. Results

### 3.1. Membrane Characterization

#### 3.1.1. Production, Ion-Exchange Capacity, and Conductivity of Cationic Membranes

The literature [39] indicates that, for cationic membranes, ion-exchange capacity is associated with the number of sulfonic groups available in the membrane. Membranes with a high degree of sulfonation (SD) usually present high ion-exchange capacity (IEC), since the concentration and mobility of ions in the membrane increase as the degree of sulfonation increases, causing conductivity also to increase [40]. The results in Table 3 indicate that the variation in the PPO:H_2_SO_4_ molar ratio and the reaction time employed in this work did not influence the SD and IEC of the membranes produced, since the three CATPPO membranes presented similar IEC and SD values. It is believed that these results are due to the sulfonation conditions employed. The sulfonation temperature of 55 °C allowed structurally stable membranes to be obtained, but the obtained SD did not exceed 20% even with 2 h of reaction. The same result was obtained by Yu and Xu [11].

In the literature [41,42], the use of concentrated sulfuric acid is reported as a sulfonating agent, but at temperatures of 250 to 350 °C. These membranes were produced to be used in energy production systems and the authors [41,42] were searching for the highest IEC possible. But the membranes were generated with structural problems, caused, among other reasons, by the high SD achieved. On the other hand, in this work, the main objective was to produce structurally stable membranes that could be used in an electrodialysis system. The use of 55 °C for sulfonation produced stable cationic membranes, reducing energy consumption in membrane production, and making the reaction conditions safer. Another important aspect of opting for sulfuric acid is its lower cost compared to other sulfonating agents (such as CSA and fuming sulfuric acid), and its availability for industrial acquisition. Table 3 presents the cationic membrane characterization results obtained according to the PPO:H_2_SO_4_ molar ratio.

Regarding the conductivity results of the cationic membranes produced in this work, it was observed that the obtained conductivity was higher than that of the membranes produced by Yu and Xu [11], where only SPPO was used. However, when compared to the conductivity present with the use of graphene oxide [30], it is observed that the conductivity of the membranes produced in this work (with only SPPO) was lower. Conductivity is an important parameter for evaluating ion-selective membranes, as it directly impacts the performance and energy consumption of the electrodialysis process [43]. Comparing the conductivity values of HDX100 and CATPPO membranes, the produced membranes presented a higher conductivity. In the case of ED application in the treatment of electroplating effluents, the physicochemical characteristics (ionic conductivity) of the effluents allow the economical application of ED using ion-selective membranes with conductivity in the same order of magnitude as found in this work [23].

#### 3.1.2. Production, Ion-Exchange Capacity, and Conductivity of Anionic Membranes

**Figure 2:**
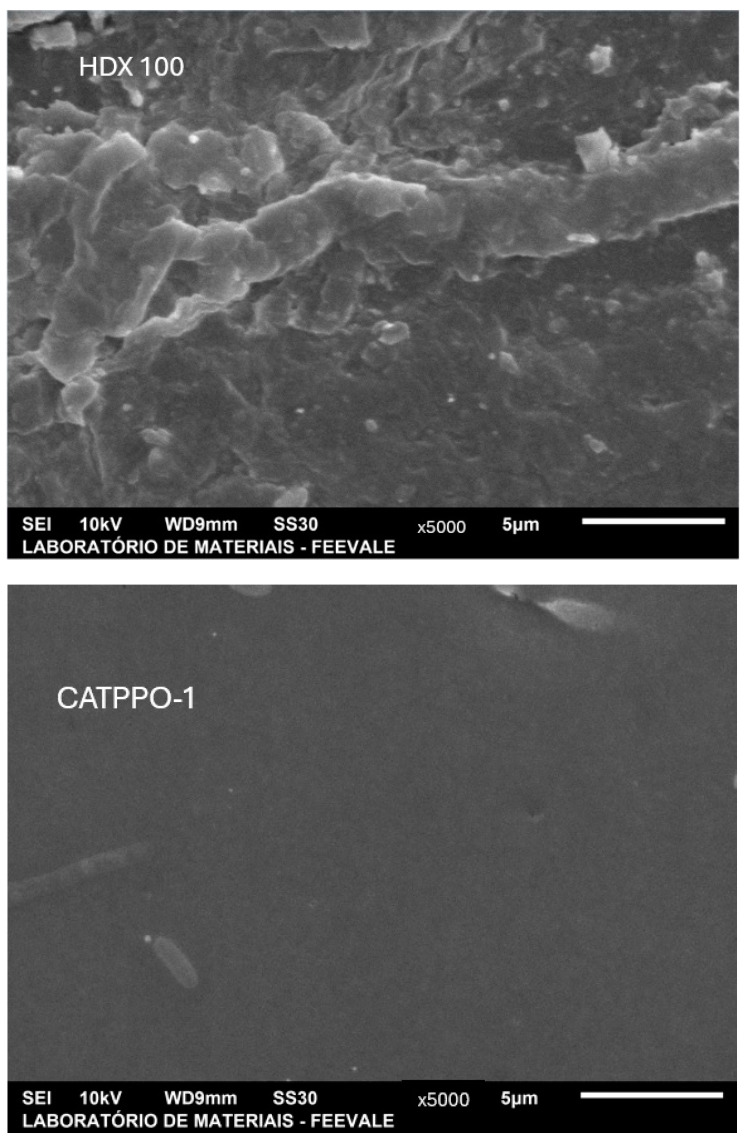
SEM surface images of the cationic membranes HDX 100 and CATPPO-1.

Regarding the production of anionic membranes, it was carried out by brominating PPO using free bromine obtained from NBS, resulting in benzyl bromide or aryl bromide groups (as shown in Figure 2). After bromination, the benzyl bromide groups will, in turn, react with amination agents, typically used in the functionalization of anionic membranes [44]. The use of bromine in the production of anionic membranes with PPO allows a short reaction time due to easy conversion with trimethylamine (TMA), through an SN2 mechanism, since bromine is an excellent leaving group [45].

From the point of view of the mechanical properties, the membranes prepared with bromination were fragile and difficult to manage, as was already cited in the literature [21]. The structural reinforcement of the anionic membranes was carried out with two types of fabrics: one based on polyamide and the other based on polyester. The membranes with polyamide fabric presented ripples. With the polyester-based fabric, the membranes were smooth and without ripples. In this study, it was observed that structural reinforcement with a woven fabric (in this case, woven polyester) is essential for anionic membrane production. Membranes without a woven fabric were tested in electrodialysis and showed dimensional instability due to excessive swelling, leading to these samples being disregarded in the subsequent stages of the investigation.

The characterization of anionic membranes, produced from bromination with two different PPO:NBS molar ratios and reinforced with woven polyester (with 14 days of amination), is presented in Table 4, which shows IEC and conductivity data for these membranes.

Regarding the IEC of the produced anionic membranes, it can be observed that the ANIPPO-1 membrane presented a higher IEC when compared to the ANIPPO-2 membrane. The higher concentration of NBS promotes a greater presence of radical species in the reaction medium, which can lead to undesirable side reactions, such as crosslinking or oxidation, and some bromination of the aromatic ring, thus decreasing the IEC [46]. Regarding the conductivity of the produced anionic membranes, it was observed that as the PPO:NBS molar ratio decreases, the conductivity of the anionic membranes increases. These results agree with those of Arges et al. [30], indicating that higher conductivity is related to higher ion-exchange capacity. The conductivity values of HDX200 and ANIPPO-1 membranes, of 2.96 × 10^−6^ mS/cm and 2.24 × 10^−6^ mS/cm, respectively, are of the same order of magnitude.

#### 3.1.3. Surface Morphology

According to the SEM investigation of the cationic membranes, the CATPPO-1 membrane showed a dense, smooth, and regular morphology, as expected from homogeneous membranes, while the HDX100 membrane showed a quite uneven surface, with roughness and many irregularities on the surface, probably due to the high amount of ion-exchange resin present, generating resin clusters in some points, as expected from a heterogeneous membrane [46,47]. These results can be seen in Figure 2, which shows SEM images of the surface of the cationic membranes HDX100 and CATPPO-1.

**Figure 3:**
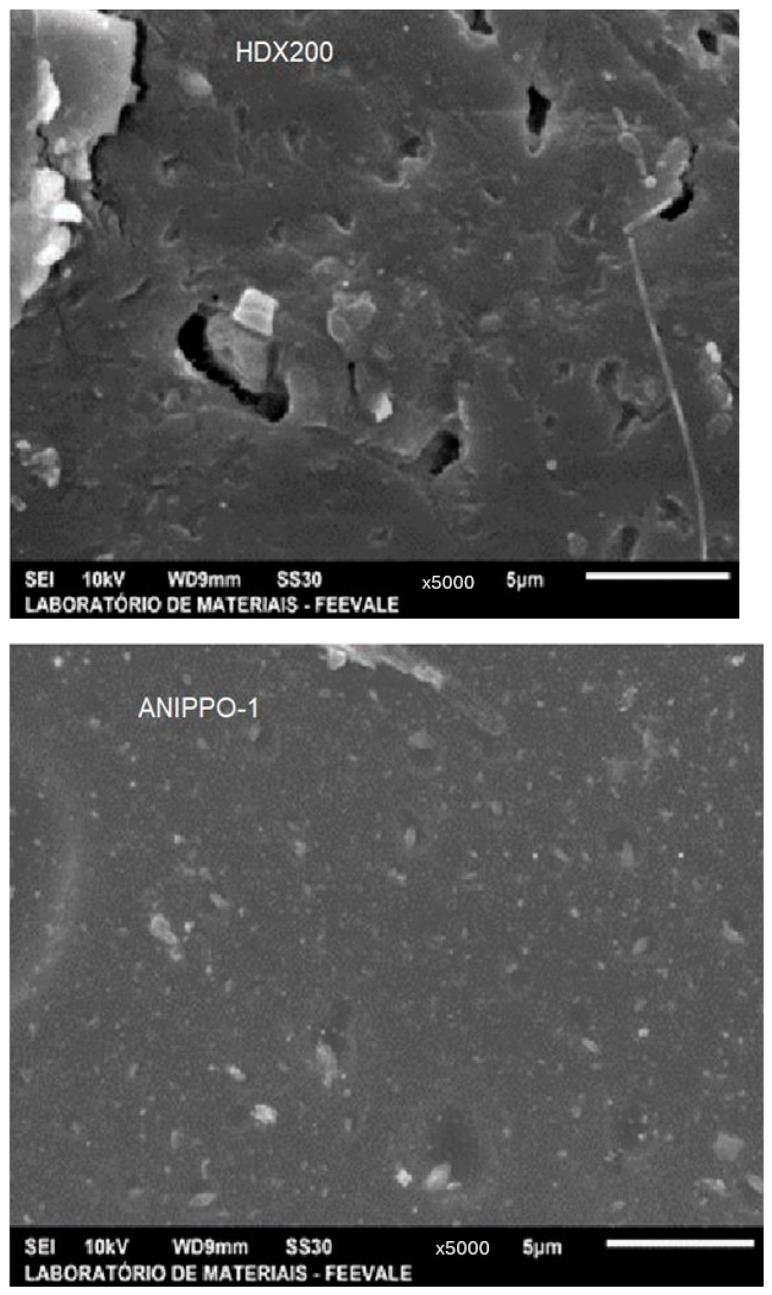
SEM surface images of the anionic membranes HDX200 and ANIPPO-1.

With regard to the anionic membranes’ morphology, although the ANIPPO-1 anionic membrane had many cavities on its surface, it presented a smoother and more uniform morphology in comparison to the HDX200 membrane which presented a heterogeneous morphology, with inserted particles in the matrix, showing surface imperfections, with many cavities, cracks, and roughness (see Figure 3).

#### 3.1.4. Thermal Stability

The thermogravimetric analysis curves of commercial and obtained cationic membranes are shown in Figure S1 in the Supplementary Material for the CATPPO-1 and HDX100 membranes. The CATPPO-1 membrane presented a mass loss of about 3% in the temperature range from 25 °C to 150 °C, with a maximum peak at 50.19 °C, related to bound water that is adsorbed by polar sulfonic groups. The intensity of this peak is an indicator of the exchange capacity of the membrane. The more water is lost (TGA), the higher the ion-exchange capacity. The HDX100 membrane presented a mass loss of about 4% in the temperature range from 25 °C to 100 °C, with a maximum peak at 83.26 °C, related to bound water [38,48,49].

A similar behavior was observed in the mass change in anionic membranes (Figure S2 in the Supplementary Material). The ANIPPO membrane presented a mass loss of about 8% in the temperature range from 25 °C to 150 °C, with a maximum peak at 64.37 °C, related to bound water. The HDX200 membrane showed a mass loss of about 4% in the temperature range from 25 °C to 100 °C, with a maximum peak at 79.33 °C, related to bound water [50].

In summary, the TGA results revealed that PPO-based cationic and anionic membranes meet the thermal stability requirements for use in electrodialysis applications, which are usually operated at room temperature or at the highest temperature of 50 °C [51].

### 3.2. Electrodialysis Experiments

#### 3.2.1. Current–Voltage Curves

For the application of ED in the recovery of nickel from effluents, it is important to determine the electric current to be used to avoid the formation of nickel precipitates on the membrane surfaces. The electric current also has an influence on the energy consumption of the treatment. The determination of the electric current to be applied was made from the results determined from CVC. To compare the results, CVC was carried out with Na_2_SO_4_ solution (0.0123 mol.L^−1^) or with nickel industrial effluent (characteristics in Table 2).

**Figure 4:**
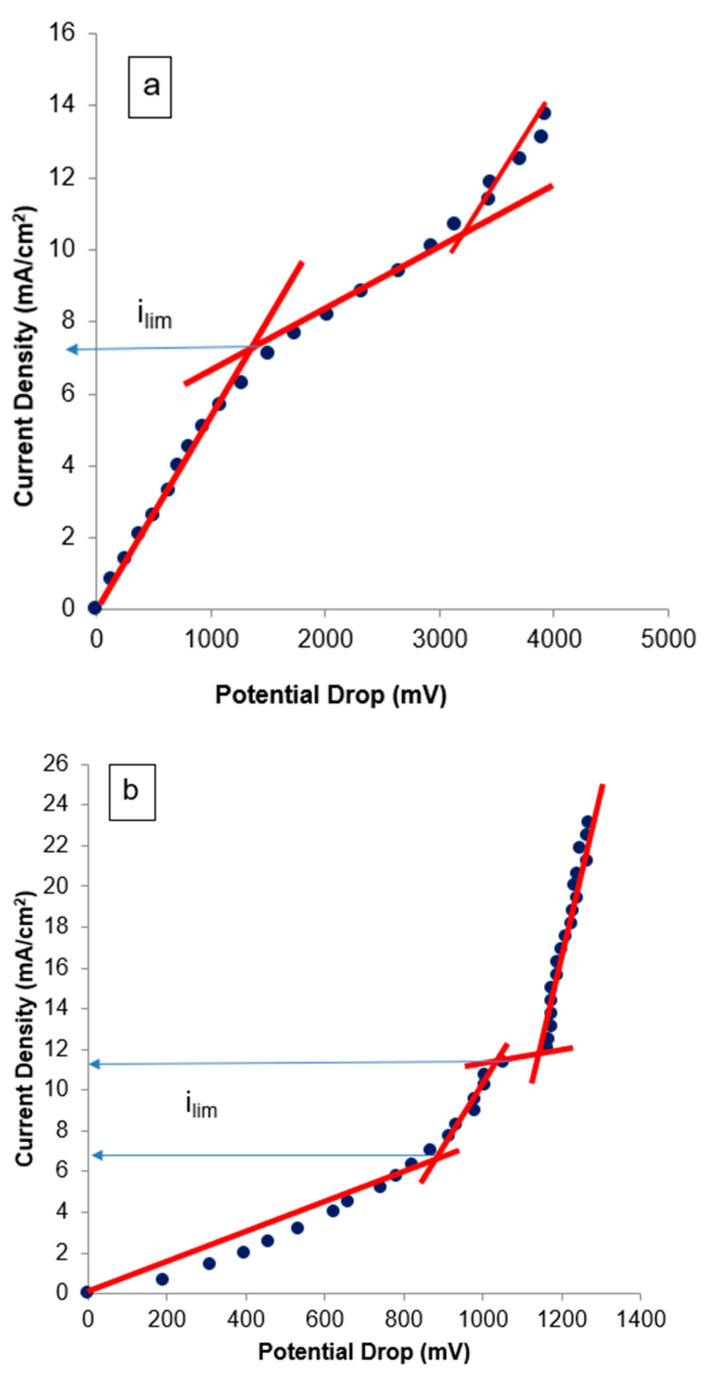

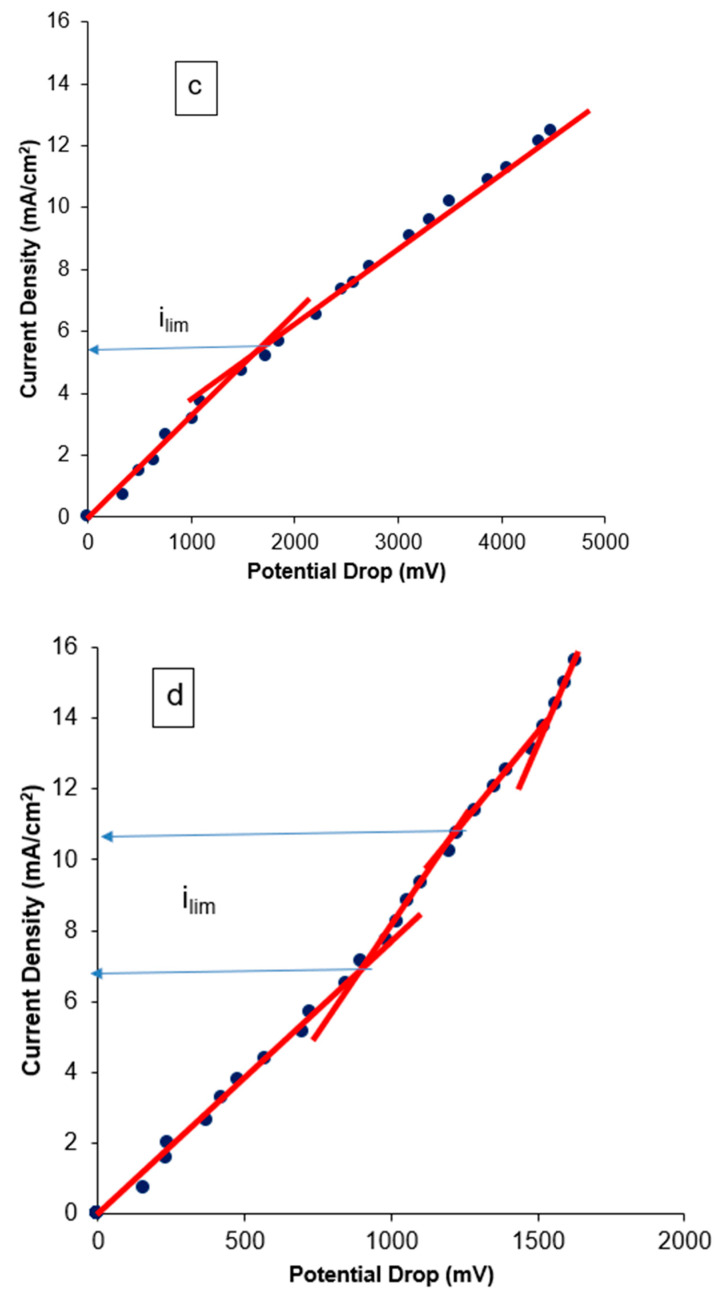
Current–voltage curves of CATPPO-1 membrane in sodium sulfate solution (**a**) or nickel industrial effluent (**b**) and of HDX100 membrane in sodium sulfate solution (**c**) or nickel industrial effluent (**d**).

Figure 4 presents the CVC for the CATPPO-1 membrane and the commercial HDX100 membrane. It can be observed that for experiments conducted with Na_2_SO_4_ and the CATPPO-1 membrane (Figure 4a), three characteristic zones were determined: quasi-ohmic, plateau, and overlimiting region. The value of the limiting current density, estimated by intercepting the ohmic and plateau regions [26], was 7.5 mA.cm^−2^. When the CATPPO-1 membrane was used with the nickel effluent (Figure 4b), two values of limiting currents could be inferred, which may be associated with the transport of different cations (Ni^+2^ and NiOH^+^) through the membranes [26]. Different authors have also obtained CVCs with two limiting current densities, and with shapes differing from the typical ones, when working with solutions with ion transport competition between two species [52,53,54,55].

The smallest value obtained in the CVC presented in Figure 4 for the CATPPO-1 membrane was 7.7 mA.cm^−2^. As the limiting current density value indicates the condition in which coupled phenomena of concentration polarization (e.g., water split) begin, and it is recommended to apply a current at about 80% of the limiting value [2], it was decided to carry out the percent extraction tests at the current density of 6.25 mA.cm^−2^, in a way that the system could operate below the limiting current, avoiding unwanted effects.

When the experiments were conducted with the HDX100 membrane (Figure 4c,d), the determined limiting current density (i_lim_) was slightly smaller, which is in accordance with the literature [26], that indicates that heterogeneous membranes usually present smaller i_lim_ than homogeneous ones. Comparing Figure 4b and Figure 4d, the shape of the CVC is the same for both membranes in nickel industrial effluent, although in the case of the HDX100 membrane, the slope of the plate in the vicinity of 10–13 mA.cm^−2^ is less pronounced.

**Figure 5:**
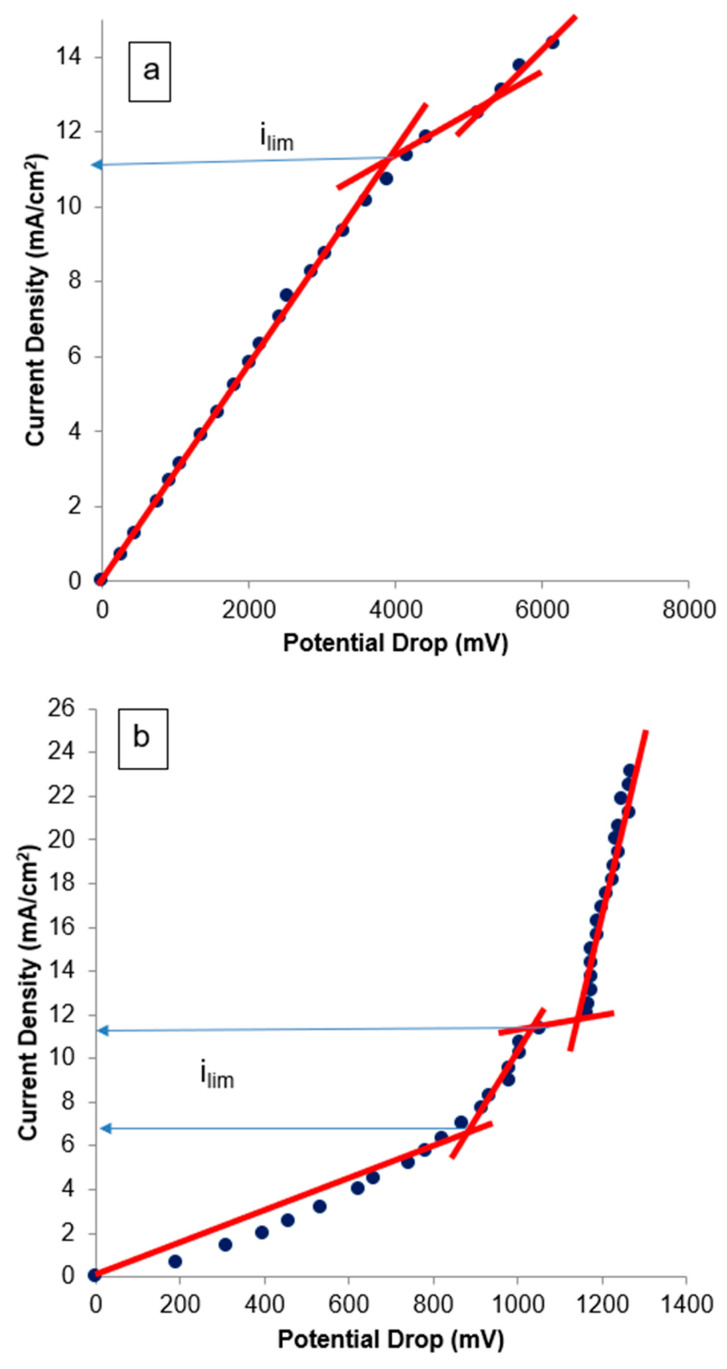

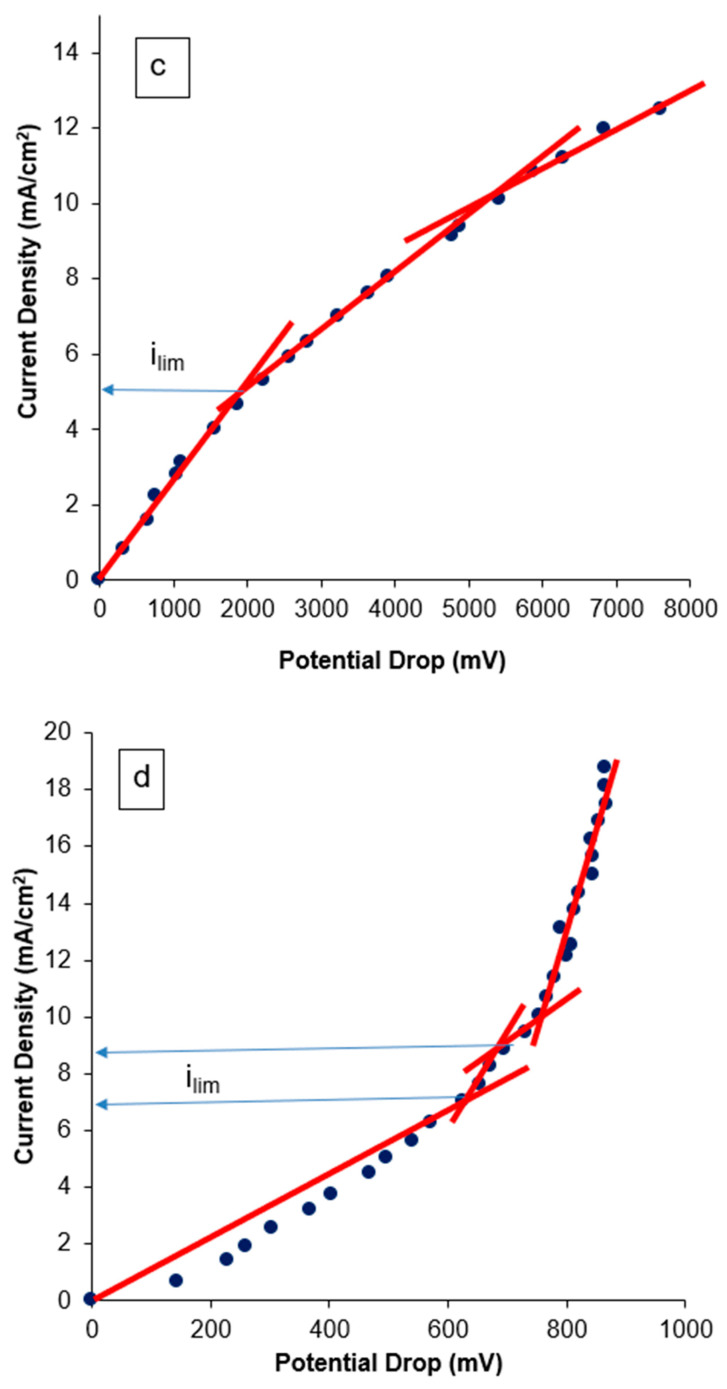
Current–voltage curves of ANIPPO-1 membrane in sodium sulfate solution (**a**) or nickel industrial effluent (**b**) and of HDX200 membrane in sodium sulfate solution (**c**) or nickel industrial effluent (**d**).

CVC experiments were also conducted for the anionic membranes ANIPPO-1 and HDX200 with sodium sulfate and with nickel industrial effluent (Figure 5).

The obtained results, presented in Figure 5, showed that the behavior of the membranes was similar to the one observed with the cationic membranes. Here, also, when Figure 5b and Figure 5d are compared, the shape of the CVC is the same for both membranes in nickel industrial effluent. By using the ANIPPO-1 and HDX200 membranes with the nickel effluent (Figure 5b,d), two values of limiting currents were also inferred. This result may be associated with the transport of different anions (SO_4_^2−^ and HSO_4_^−^) through the membranes [26,52]. The ionic transport in a nickel sulfate solution was studied by Benvenuti et al. [26], who also studied an industrial effluent with the same characteristics as the one evaluated at this work. Using chronopotentiometry, the authors [26] detected the transport of HSO_4_^−^ and SO_4_^2−^ ions, and considered that, since the concentration of the nickel–sulfate complex [Ni(SO_4_)_2_]^2−^ is considerably lower than HSO_4_^−^, the species [Ni(SO_4_)_2_]^2−^ may be transported together with HSO_4_^−^.

The experiments conducted with the HDX200 membrane (Figure 5c,d) also resulted in a smaller limiting current density, when compared to the ones obtained with ANIPPO-1.

The values obtained by the CVC of the anionic membranes are of the same magnitude as the ones obtained with the cationic membranes, confirming the value of 6.25 mA.cm^−2^ as a good choice for further experiments.

The CVC results demonstrate that the limiting current densities for the produced membranes are higher than the one for the commercial membranes, indicating that the synthesis conditions of the cationic and anionic membranes used in this work were suitable for producing membranes to be used in ED.

#### 3.2.2. Ion Extraction by Electrodialysis

The results of nickel and sodium extraction by the CATPPO-1 membrane compared to the commercial HDX100 membrane are presented in Table 5. It can be observed that the CATPPO-1 membrane shows a higher extraction of nickel and sodium compared to the commercial membrane, suggesting that the membrane synthesis was adequate. When relating these results to the sulfonation conditions investigated, it is observed that, with a 20% sulfonation degree, the membrane already shows a high extraction of nickel and sodium. Therefore, for the development of cationic membranes to be applied in ED, sulfuric acid at 55 °C can be used as a sulfonating agent.

According to the values presented in Table 5, the ED test with nickel effluent and the HDX200 membrane resulted in a sulfate content reduction of 76.5%. In comparison, the sulfate content reduction for the ANIPPO-1 membrane was 90.9%. Similarly, a greater efficiency in reducing chloride ion content was observed for the ANIPPO-1 membrane. The results of the removal of sulfate and chloride ions by the ANIPPO-1 membrane allow the evaluation that the reaction conditions were able to produce membranes efficient in ion extraction. It is important to highlight that under the experimental conditions applied in this work, no nickel deposit was observed on the cationic or anionic membranes.

## 4. Conclusions

The procedures for obtaining cationic membranes using only concentrated sulfuric acid at 55 °C for the sulfonation of poly(2,6-dimethyl-1,4-phenylene oxide) resulted in a sulfonation degree of 20%. These membranes exhibited excellent structural behavior and high efficiency in extracting sodium and nickel ions. This efficiency makes them suitable for nickel effluent treatment with a focus on zero effluent disposal and nickel ion recovery, thereby promoting the circular economy.

The anionic membranes produced showed no significant variations in the analyzed parameters as a function of the PPO:NBS molar ratio. Anionic membranes without woven reinforcement were unstable, highlighting the necessity of such reinforcement. This requirement contrasts with the behavior of the cationic membranes, which did not need woven reinforcement.

Overall, PPO demonstrates significant potential as an industrial polymer for the preparation of both anionic and cationic ion-selective membranes. This research underscores the importance of structural reinforcement in anionic membranes and the robustness of cationic membranes without such reinforcement, paving the way for advancements in effluent treatment and resource recovery.

## Figures and Tables

**Table 1:** Main characteristics of the commercial cation-exchange (HDX100) and anion-exchange (HDX200) membranes provided by the supplier [22].

Parameter	HDX100	HDX200	Unit
Ionic group attached	-SO_3_^−^	-NR_3_^+^	--
Water content	35–50	30–45	%
Ion-exchange capacity	≥2.0	≥1.8	mol/kg (dry)
Membrane surface resistance (0.1 mol NaCl)	≤20	≤20	Ω cm^2^
Permselectivity (0.1 mol KCl/0.2 mol KCl)	≥90	≥89	%
Burst strength	≥0.6	≥0.6	MPa
Dimension change rate (longitudinal and lateral)	≤2	≤2	%
Water flux	≤0.1 (below 0.2 MPa)	≤0.2 (below 0.035 MPa)	mL/h·cm^2^

**Table 2:** Industrial effluent characteristics.

Nickel	10.2 mmol.L^−1^
Sodium	123.5 mmol.L^−1^
Sulfate	29.6 mmol.L^−1^
Chloride	16.8 mmol.L^−1^
Conductivity (25 °C)	3 mS.cm^−1^
pH	4.4

**Table 3:** PPO:H_2_SO_4_ molar ratio, reaction time, sulfonation degree, ion-exchange capacity, and conductivity of obtained cationic membranes compared to the literature and the commercial membrane HDX100.

Membrane	PPO:H_2_SO_4_ Molar Ratio	Reaction Time (h)	SD (%)	IEC (meq/g)	Conductivity (mS/cm) (25 °C)
CATPPO-1	1:4.4	0.5	20.80	1.52 ± 0.012	15.3
CATPPO-2	1:4.4	2.0	19.93	1.47 ± 0.003	13.0
CATPPO-3	1:5.5	0.5	19.93	1.47 ± 0.002	13.0
SPPO (ref. [11])	-	-	17.2	1.28	0.57
SPPO-FGO (7%) (ref. [30])	-	-		1.76	100
HDX100 (ref. [23])				2.4	5.45

**Table 4:** PPO:NBS molar ratio, ion-exchange capacity, and conductivity of the obtained anionic membranes compared to the commercial membrane HDX200.

Membrane	PPO:NBS Molar Ratio	IEC (meq/g)	Conductivity (mS/cm) (25 °C)
ANIPPO-1	1:0.5	1.88 ± 0.029	2.24
ANIPPO-2	1:0.7	1.07 ± 0.011	0.605
HDX200 (ref. [23])	-	1.67	2.96

**Table 5:** Percent extraction of ions from the nickel industrial effluent by electrodialysis with the produced and commercial membranes (2 h at 6.25 mA/cm^2^).

Membranes	Nickel PE (%)	Sodium PE (%)	Sulfate PE (%)	Chloride PE (%)
CATPPO-1 + HDX200	85.5	41.3	n.d.	n.d.
HDX100 + HDX200	55.4	39.6	76.5	77.2
ANIPPO-1 + HDX100	n.d	n.d.	90.9	91.8

n.d. = not determined.

## Data Availability

The data that support the findings of this study are available from the corresponding author upon reasonable request.

## Supplementary Materials

The following supporting information can be downloaded at: https://www.mdpi.com/article/10.3390/membranes14120268/s1, Figure S1. Thermogravimetric analysis curves of commercial membrane HDX 100 and obtained membrane CATPPO-1; Figure S2. Thermogravimetric analysis curves of commercial membrane HDX200 and obtained membrane ANIPPO-1. References [10,38,48,49,50,51,56,57,58,59,60,61,62] are cited in the supplementary materials.
