# Supplementary material for: Cationic/Anionic Poly(p-Phenylene Oxide) Membranes: Preparation and Electrodialysis Performance for Nickel Recovery from Industrial Effluents
Source: Membranes (Basel). 2024 Dec 11;14(12):268. doi: 10.3390/membranes14120268 (PMC11678474; doi:10.3390/membranes14120268)
Supplement: Supplementary file 1 [file membranes-14-00268-s001.zip › membranes-3313928-supplementary.pdf]

SUPPLEMENTARY MATERIAL

Cationic/anionic poly(p-phenylene oxide) membranes: preparation and electrodialysis performance for nickel recovery from industrial effluents

F. Wilbert [1], J. F. Corte [1], F. T. do Nascimento [1], V. D. Jahno [1], M. A. S. Rodrigues [1], F. Celso [2], S. W. da Silva [3] and A. M. Bernardes [4*]

[1] Laboratório Aquário, Novo Hamburgo, Feevale University, Brazil;
[2] Development Technology Center, Federal University of Pelotas, Pelotas, Brazil;
[3] IPH, PPGRHS - Federal University of Rio Grande do Sul, Porto Alegre, Brazil;
[4] PPGE3M - Federal University of Rio Grande do Sul, Porto Alegre, Brazil;
[*] Corresponding author: Andrea Moura Bernardes: amb@ufrgs.br

Thermal stability

Thermogravimetric analysis curves of commercial and obtained cationic membranes are showed in Figure S1 for CATPPO-1 and HDX100 membranes.

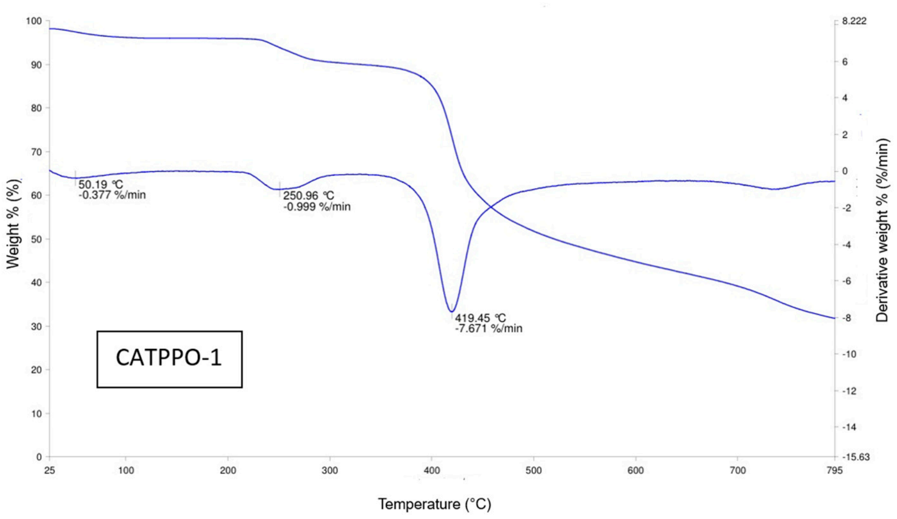

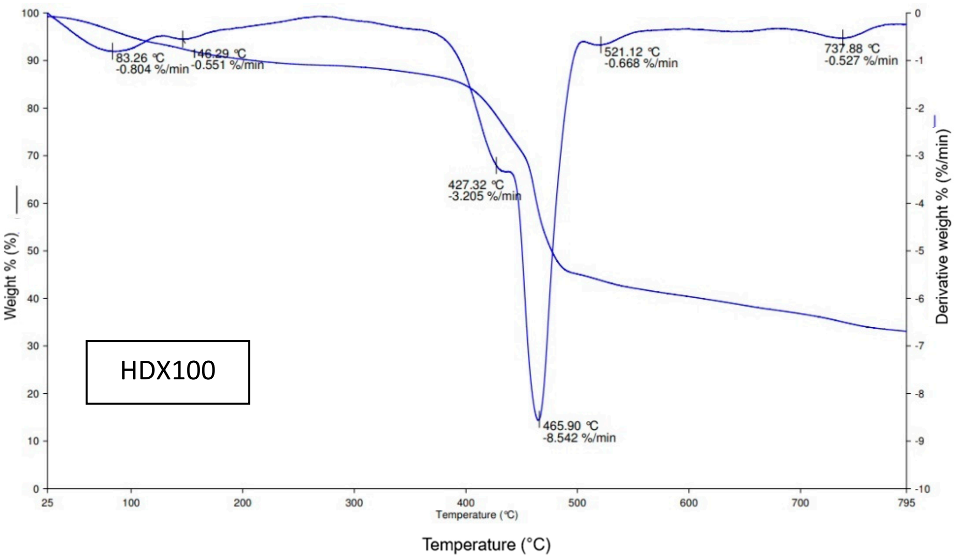

Figure S1. – Thermogravimetric analysis curves of HDX 100 commercial membrane and CATPPO-1 obtained membrane.

As can be seen from the TGA and DTG shown in Figure S1, referring to the evaluated cationic membranes, the mass change of membrane samples occurred via three main stages for the homogeneous CATPPO membrane, and via four main stages for the heterogeneous HDX100 membrane.

CATPPO-1 membrane presented a mass loss of about 3% in the first step, ranging from 25°C to 150°C with a maximum peak at 50.19°C, related to bound water that is adsorbed by polar sulfonic groups. The intensity of this peak is an indicator of the exchange capacity of the membrane. The more water is lost (TGA), the higher the ion exchange capacity. A second step, ranging from 200°C to 350°C with a maximum peak at 250.96°C, is related to sulfonic acid groups degradation, and a third step, ranging from 350°C to 800°C with a maximum peak at 419.45°C, is related to PPO backbone degradation [1, 2]. Similar behavior is reported for SPPO-based membranes [3 - 5].

In a more complex behavior, HDX100 membrane presented a mass loss of about 4% in the first step, ranging from 25°C to 100°C with a maximum peak at 83.26°C, related to bound water. A second step, ranging from 100°C to 300°C with a maximum peak at 146.29°C, is related to sulfonic acid -SO_3^- group degradation, a third step, ranging from 300°C to 450°C with a maximum peak at 427.32°C, is related to polyamide reinforcement mesh degradation, and a fourth step, ranging from 450°C to 500°C, with a maximum peak of 465.90°C, is related to the polymeric high density poly(ethylene) matrix backbone degradation [6].

A similar behavior was observed in the mass change of anionic membranes. i.e., three main stages of mass change for the ANIPPO homogeneous membrane, and four main stages of mass change for the HDX200 heterogeneous membrane (Figure S2).

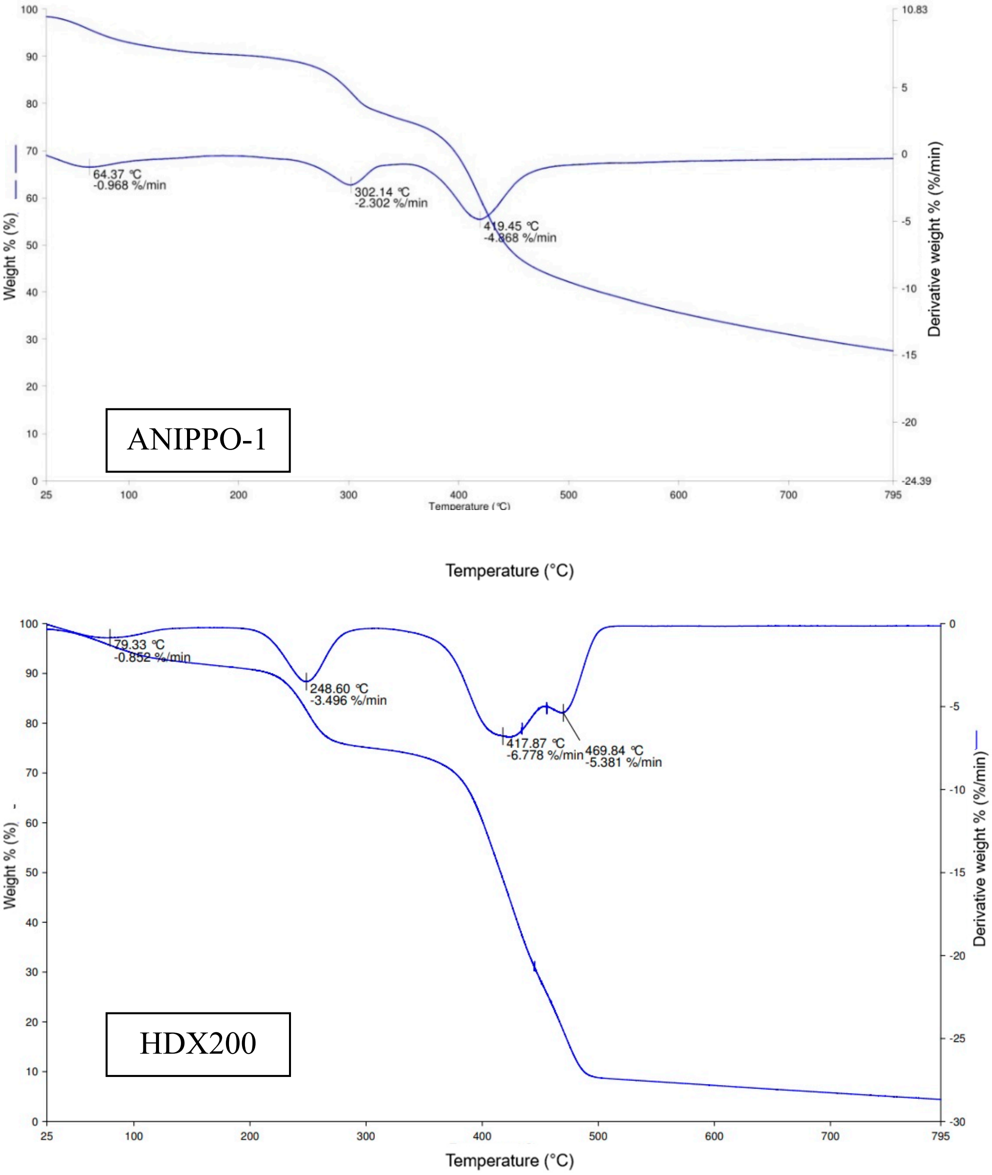

Figure S2. – Thermogravimetric analysis curves of commercial membrane HDX200 and obtained membrane ANIPPO-1.

ANIPPO membrane presented a mass loss of about 8% in the first step, ranging from 25°C to 150°C with a maximum peak at 64.37°C, related to bound water. A second step, ranging from 200°C to 350°C with a maximum peak at 302.14°C, is related to amine groups degradation, and a third step, ranging from 350°C to 800°C with a maximum peak at 419.45°C, is related to PPO backbone degradation. Similar behavior was reported for BPPO-based anion exchange membranes [7 - 10].

Similar to cationic HDX100 membrane, HDX200 membrane showed a more complex behavior, with a mass loss of about 4% in the first step, ranging from 25°C to 100°C with a maximum peak at 79.33°C, related to bound water [11]. A second step, ranging from 150°C to 350°C with a maximum peak at 248.60°C, is related to quaternary amine functional groups $-NR_3^+$ degradation [12, 13], a third step, ranging from 350°C to 450°C with a maximum peak at 417.87°C, is related to polyamide reinforcement mesh degradation,

and a fourth step, ranging from 450°C to 550°C with a maximum peak of 469.84°C, is related to the polymeric high density poly(ethylene) matrix backbone degradation [6].

In summary, TGA results revealed that PPO cationic and anionic based membranes meet the thermal stability requirements for being used in electrodialysis applications, which are usually operated at room temperature, or at the highest temperature of 50°C [10].

References

1. Feng, Z.; Esteban, P.; Gupta, G.; Fulton, D,; Mamlouk, M. Highly conductive partially cross-linked poly(2,6-dimethyl-1,4-phenylene oxide) as anion exchange membrane and ionomer for water electrolysis. Intern. J. Hydr. Energy. 2021, 46, 37137-37151.
2. Ong, A. L.; Saad, S.; Lan, R.; Goodfellow, R. J.; Tao, S.; Anionic membrane and ionomer based on poly(2,6-dimethyl-1,4- phenylene oxide) for alkaline membrane fuel cells. Journal of Power Sources, 2011, 196, 20, 8272–8279.
3. Suhag, S.; Kumar, P.; Mandal, J.R.; Shahi, V.K.; Functionalized graphene oxide-modified sulfonated poly (2,6-dimethyl-1,4-phenylene oxide) based thermal-resistance anti-fouling bi-functional cation exchange membrane for electrodialytic desalination, Desalination, 2024, 578, 117454.
4. Yang, S.; Gong, C.; Guan, R.; Zou, H.; Dai, H.; Sulfonated poly(phenylene oxide) membranes as promising materials for new proton exchange membranes. Polymers for Advanced Technologies, 2006, 17, 360-365.
5. Petreanu, I.; Marinoiu, S.; Sisu, C.; Varlam, M.; Fierascu, R.; Stanescu, P.; Teodorescu, M.; Synthesis and testing of a composite membrane based on sulfonated polyphenylene oxide and sílica compounds as proton exchange membrane PEM fuel cells. Materials Research Bulletin, Volume 96, Pages 136-142 (2017)].
6. Feijoo, G.G.; Barros, K.S.; Scarazzato, T.; Espinosa, D.C.R.; Electrodialisys for concentrating cobalt, chromium, manganese, and magnesium from a synthetic solution based on a nickel laterite processing route. Separation and Purification Technology, 2021, 275, 119192.
7. Khan, M.I.; Mondal, A.N.; Tong, B.; Jiang, C.; Emmanuel, K.; Yang, Z.; Wu, L.; Xu, T.; Development of BPPO-based anion exchange membranes for electrodialysis desalination applications. Desalination, 2016, 391, 61-68.

8. Rathod, N.; Yadav, V.; Rajput, R.; Sharma, J.; Shukla, D.K.; Kulshresha, V.; New class of composite anion exchange membranes based on Quaternized poly(phenylene oxide) and functionalized boron nitride. Colloid and Interface Science Communications, 2020, 336, 100265.
9. Lee, S.B.; Min, C.M.; Jang, J.; Lee, J.S.; Enhanced conductivity and stability of anion exchange membranes depending on chain lenghts with crosslinking based on poly(phenylene oxide). Polymer, 2020, 192, 122331.
10. Khan, M. I.; Khraisheh, M.; Almonani, F. Innovative BPPO Anion Exchange Membranes Formulation Using Diffusion Dialysis-Enhanced Acid Regeneration System. Membranes 2021, 11, 1-17.
11. Afsar, N. U.; Ji. W.; Wu, B.; Shehzad, M. A.; Ge, L.; Xu, T. . SPPO-based cation exchange membranes with a positively charged layer for cation fractionation. Desalination 2019, 472, 114145.
12. Gubari, M.Q.; Zwain, H.M.; Alekseeva, N.V.; Baziyani, G.I.; Features of feed concentration and temperature effects on membranes operation in electrodialysis systems – a review. Journal of Physics: Conference Series, 1973, 012178 (2021).
13. Jiang, Y.; Liao, J.; Yang, S.; Li, J. Stable cycloaliphatic quaternary ammonium-tethered anion exchange membranes for electrodialysis. Reac. Func. Polym. 2018, 130, 61-69.
